# Primary perivascular epithelioid cell tumor of the liver: new case report and literature review

**DOI:** 10.1186/1746-1596-9-149

**Published:** 2014-07-17

**Authors:** Hassania Ameurtesse, Laïla Chbani, Amal Bennani, Imane Toughrai, Nouhad Beggui, Imane Kamaoui, Hinde Elfatemi, Taoufik Harmouch, Afaf Amarti

**Affiliations:** 1 Department of Pathology, Hassan II University Hospital, Fez, Morocco; 2 Department of surgery, Hassan II University Hospital, Fez, Morocco; 3 Department of Radiology, Hassan II University Hospital, Fez, Morocco

**Keywords:** PEComas, Liver neoplasms, Perivascular epithelioid cell

## Abstract

Perivascular epithelioid cell tumors (PEComas) encompass a group of rare mesenchymal neoplasms, which typically have a perivascular location with dual melanocytic and muscular differentiation. They are found in a variety of localizations, though lesions in the liver are exceedingly rare. Because of their rarity, the clinical, radiological and histological features of these tumors have yet to be established. This is why, it seems appropriate to report the observation of this rare hepatic tumor with a literary review including others published cases, assessing through it, clinicopathologic and radiologic features of all reported cases as well as their follow-up whenever possible.

**Virtual Slides:**

The virtual slide(s) for this article can be found here: http://www.diagnosticpathology.diagnomx.eu/vs/1967094999126169

## Background

Perivascular epithelioid cell neoplasms (PEComas) formed a rare group of related mesenchymal tumors composed of histologically and immunohistochimically distinctive perivascular cells (PECs), first proposed by Bonetti *et al*. in 1992. The members of this family include angiomyolipoma, lymphangioleiomyomatosis, pulmonary clear cell “sugar” tumors and PEComa-NOS [1]. This last entity, first introduced by Zamboni and al in 1996, to describe neoplasms composed solely of PECs in his case report [2]. Many anatomic sites can be affected, but the uterus is the more common. Cases that arise from the liver are extremely rare [1]. To the best of our knowledge, Only 20 hepatic PEComas, composed solely of PECs, have been reported in the current literature, mostly, as single-case reports.

The following report presents an additional case of primary liver PEComa appearing in a 63-years-old woman, followed by a short synopsis about previously published cases. We discuss the clinicopathological features, the immunophenotype and the differential diagnosis to improve awareness about this type of tumor and how to diagnose it.

## Case presentation

A 63-year-old woman presented to gastroenterology department with a 1-year history of atypical pain in the right upper abdominal quadrant, with significant fatigue and weight loss.

**Figure 1:**
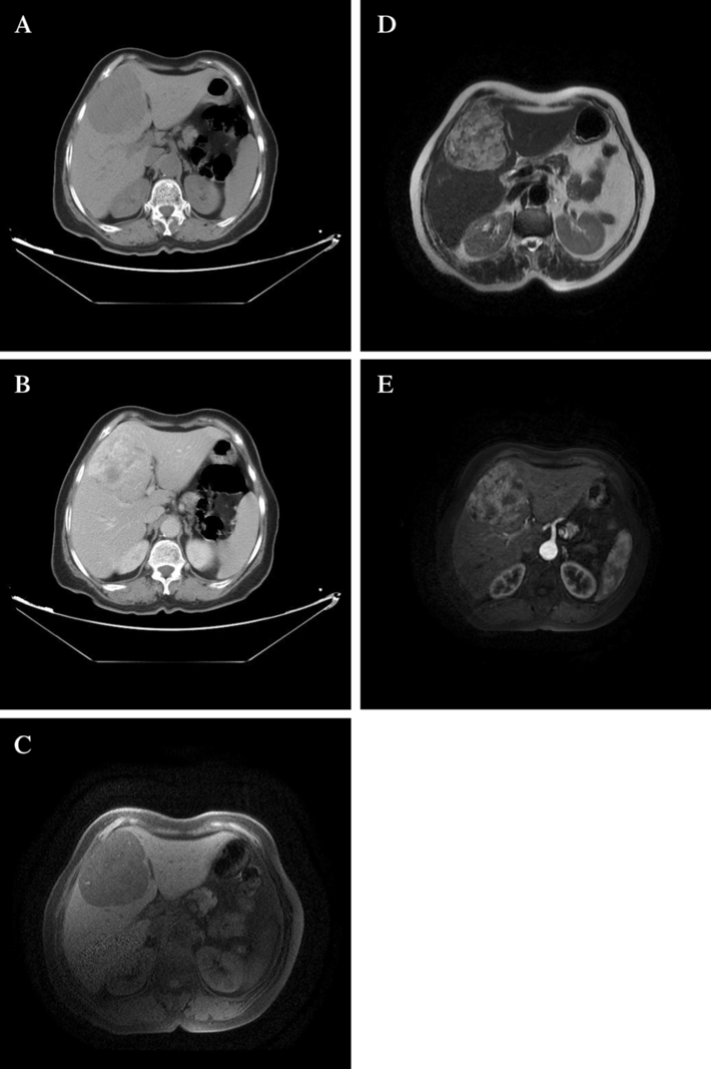
**Radiological features of the tumor. A)** precontrasted CT scan revealed a low-density mass of segment IV of the liver with ill-defined borders. **B)** Contrast CT showing inhomogeneous contrast enhancement of tumor in the portal phase. **C)** A mass described above had a low signal on T1-Weighted MRI, it became hyperintense on T2-Weighted images **(D)**, and presented a strong and heterogeneous enhancement after injection of gadolinium **(E)**.

Her past medical history was unremarkable. She had no evidence of cirrhosis, viral hepatitis or tuberous sclerosis complex (Bourneville’s disease). Physical examination revealed only mild hepatomegaly without palpable mass or jaundice. Laboratory examinations showed normal liver function tests and tumoral marquers (AFP, CEA).Initial abdominal ultrasonography revealed a solitary, heterogeneous and hypoechoic mass in the liver, with an ill-demarcated margin. Color Doppler flow images showed abundant blood flow in the marginal area of the tumour. Subsequent CT and abdominal MRI confirmed this intrahepatic mass to be located in the fourth segment (IV), measuring 6,4 × 8 cm in diameters (Figure 1: A, B, C, D and E). Neither lymphadenopathy nor portal vein involvement was present. No additional lesion was detected. Given the hypervascular character, the lack of adipose tissue and thick-walled blood vessels, the lesion has been interpreted as hemangiomas, atypical hepatocellular carcinoma or metastatic lesion. Owing to the deteriorating clinical situation and the ambiguous imaging findings, a central segmentectomy of the liver with a cholecystectomy was performed.

**Figure 2:**
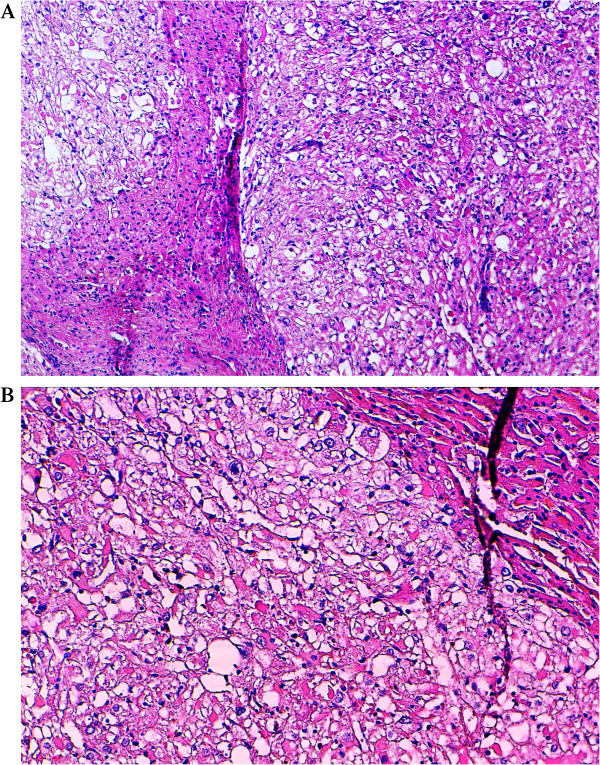
**Histopathological features of the proliferation. A)** ill-defined sheets of pleomorphic round to polygonal epithelioid cells, infiltrating adjacent liver. The cytoplasm abundant clear to eosinophilic, with distinct borders. **B)** The nuclei were round to oval with finely vesicular chromatin and small nucleoli. Focal nuclear pleomorphism was observed. H&E. A, × 100, B, × 200.

**Figure 3:**
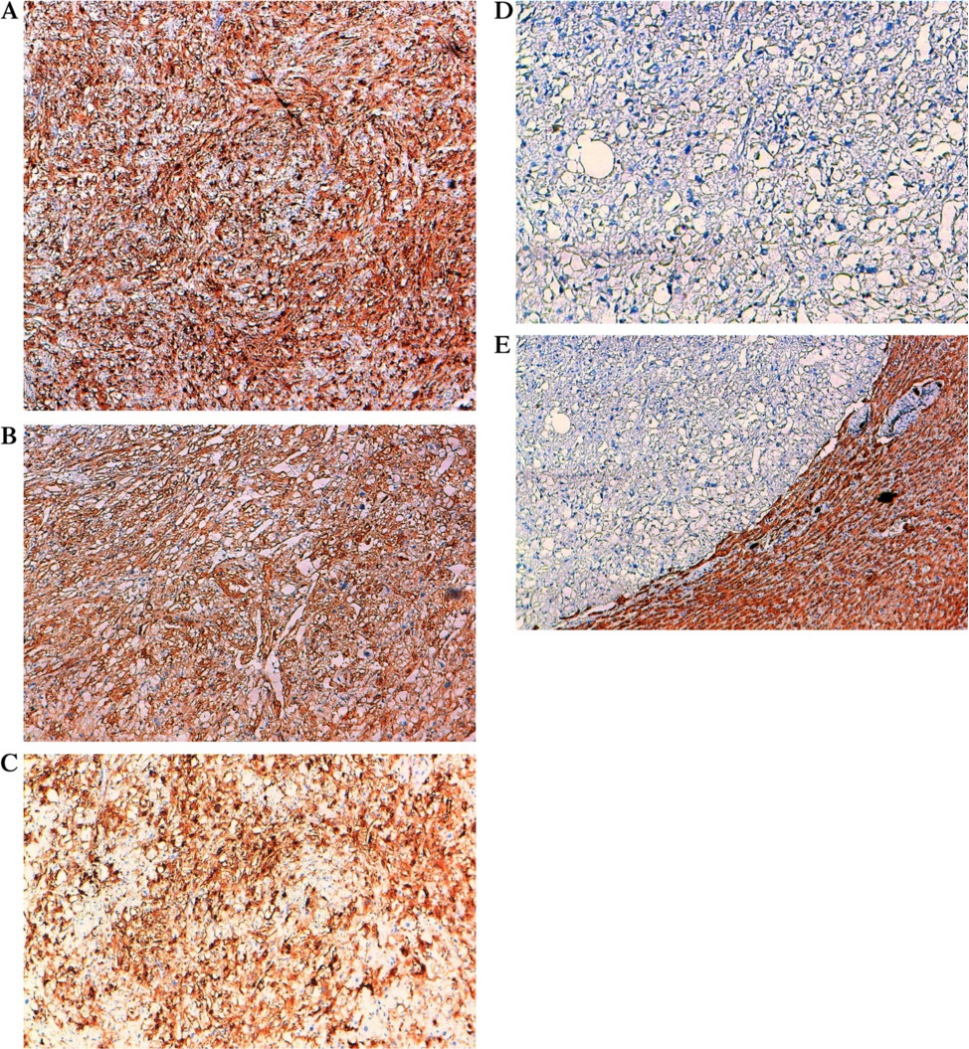
**Immunohistochemical profile of PECs.** IHC × 100 **A)** Most of epithelioid tumor cells are markedly immunoreactive for HMB45 **(A)**, and SMA **(B)**. Focal Melan-A immunopositivity was noted **(C)**. S100 protein **(D)** and Cytokeratin **(E)** were completely negative. IHC × 100.

Gross examination revealed a 6 × 8 cm, solitary lesion with an ill-demarcated margin. The external surface of the mass was smooth, white-tan in color. Cut sections through the mass showed solid and tiny cystic areas with multiples congested blood vessels. The hepatic tissues around the mass were normal. The authors took 15 sections from different parts of the tumor and 2 sections from the adjacent liver.Routine hematoxylin-eosin-stained sections from formalin-fixed, paraffin-embedded tissue ware examined. Histologically, the tumor was composed of nests and sheets of larges and round to polygonal cells, separated by a rich sinusoidal vascular network. The cells showed abundant cytoplasm that varied from eosinophilic granular to clear, with distinct cell border. The nuclei were round to oval with finely distributed to vesicular chromatin and small nucleoli. Focal nuclear pleomorphism was observed, without necrosis, mitotic figures or angiolymphatic invasion [Figure 2]. The tumor frequently showed dilated vascular channels with radial arrangement of tumor cells. In numerous sections, adipocytes, thick-walled vessels and hematopoietic cells were entirely absent. The adjacent liver was microscopically normal.Immunohistochemical study showed a strong and diffuse expression of HMB45 and smooth muscle actin (SMA), while Melan-A was focally positive. The neoplastic cells were negative for S100 protein and Pancytokeratin [Figure 3].

On the basis of the morphologic characteristics and immunohistochemical results, a diagnosis of hepatic PEComa was made, with infiltration of the margins.

The patient recovered uneventfully and was discharged 2 weeks after surgery, without any adjuvant treatment. At present, 9 months after the surgery, she is under regular clinical follow-up with no evidence of primary recurrence or metastasis.

### Bibliographic research

For the literature review, a systematic search for PEComa related reports published was performed using PubMed [3] with de keywords: PEComa, perivascular epithelioid cell, angiomyolipoma, liver and HMB45.

## Discussion

We have described a hepatic PEComa composed purely of epithelioid cells with lack of lipocytes and abnormal vessels. In our case, no other component element of the tumour could be found anywhere even though extensive examination of most of the tumour tissue was performed.

The descriptive term “perivascular epithelioid cells (PECs)” were first proposed by Bonetti *et al*. in 1992 to describe an “unusual atypical cell type” which typically has a perivascular distribution, with dual melanocytic and myoid differentiation [4]. Despite the lack of evidence of a normal anatomical homologue, in 2002, the World Health Organization has given formal recognition to the concept of this novel cell type and of PEC-derived tumors. “PEComa family” includes now many distinct clinicopathologic entities such as angiomyolipoma (AML), lymphangiomyomatosis and clear cell “sugar” tumors of the lung, [1]. Some of them have been linked to tuberous sclerosis complex, especially AML of the kidney. The group comprising solely PECs is rare; it has been discriminated from classic angiomyolipoma by various appellations including monotypic epithelioid AML, clear cell myomelanocytic tumor, primary extrapulmonary sugar tumor, and PEComas-NOS or simple PEComa [5,6]. The first reported case in the liver was in 2000, when Yamasaki S described this entity diagnosed incidentally in a 30-years-old women [7]. Since, twenty prior case reports were found in a MEDLINE search and we auditioned our case to this data, the main findings for these cases are summarized in Additional file 1: Table S1 [6-24].

Hepatic PEComa was commonly diagnosed following abdominal pain or mass, occasionally with incidental finding. One case had synchronous GIST. No one had history or symptoms of TSC. The routine laboratory investigations were noncontributory.

There was a marked female predominance (17 women and 4 men), and the mean age at diagnosis was 46.3 years (range 25–60 years). All tumors were presented as a solitary lesion, most often in the right lobe, which poses a clinical and radiological diagnostic challenge. They varied from 0.8-17 cm in greatest dimension (mean 8.1 cm).

PEComas exhibit a wide spectrum of imaging findings. Hepatic PEComas can be of any echogenecity, most often, with abundant blood flow in or surrounding the lesion. As our case, on CT scan, most neoplasms present as hypo intense with significant enhancement on arterial phase. The portal phase was variable. Almost all lesions reported show low-signal on T1-weighted images, high-signal on T2-weighted images. These radiologic findings of hypervascular lesion can be confused, most often, with those of hepatocellular carcinoma or simple haemangioma [25].

The diagnostic was most often confirmed by pathology finding. PEComa cells are characterized by their perivascular location, often with radial arrangement around the vascular lumen. Typically, tumor cells are epithelioid and spindle-shaped resembling smooth muscle cells, and tend to have abundant clear to eosinophilic pale granular cytoplasm [5]. Few cases are reported with prominent nucleoli and mild nuclear pleomorphism as seen in our case. The presence of melanin pigment is extremely rare, reported only in 3 cases [13,19,20].

The most important finding is positive immunostaining with both melanocytic (HMB45 and/or melan A) and smooth muscle (actin and/or desmin) markers [5]. In all reports, there was a diffuse positivity for HMB45. Melan A and SMA were frequently positive. S100 protein, desmin and vimentine were more often negative.

Monotypic epithelioid angiomyolipoma should be differentiated from epithelioid smooth muscle tumour. Immunohistochemically or ultrastructurally, both tumours express markers of smooth muscle differentiation, but only monotypic epithelioid angiomyolipoma expresses markers of melanogenesis. Other common differential diagnoses include hepatic adenoma and carcinoma, gastrointestinal stromal tumors, epithelioid sarcoma, paraganglioma, and metastatic sarcomatoid renal cell carcinoma as well as oncocytic and clear cell carcinoma. The positivity for melanocytic markers and the negativity for multiples markers including CK, CD117, chromogranin and synaptophysin confirmed the diagnosis [5,15] (Table 1).

**Table 1:** Immunohistochemistry in the differential diagnosis of PEComa tumors

	**PEComa**	**Gastrointestinal stromal tumors**	**Carcinoma**	**Melanoma**	**Paraganglioma**
**SMA**	positive	variable			
**H-caldesmon**	positive				
**Desmine**	positive	variable			
**HMB-45**	positive			positive	
**Melan-A**	positive			positive	
**S-100 protein**	rarely	variable		positive	positive
**Chromogranin A**	negative	rarely			positive
**Synaptophysin**	negative	rarely			positive
**CK**	negative	negative	positive	negative	
**EMA**	negative		positive	negative	
**CD117**	negative	positive			
**CD34**	negative	positive			

The possibility of metastatic malignant melanoma should always be removed. In the present case, the patient had no history or clinical manifestation of cutaneous or mucosal melanoma. All the more, we had a negative immunostaining with S100 protein. This last protein was expressed in many reported cases [7,15,19-21], thereby making the differential diagnosis more difficult.

Although the vast majority of reported PEComa showed a benign course; some are aggressive with locally destructive recurrences, and distant metastasis. To date, defined criteria for malignancy in hepatic PEComa have not been established. Folpe *et* al. [26] proposed a classification of PEComas into benign, uncertain malignant potential, and malignant based on the presence of seven worrisome histological features: Tumor size > 5 cm, infiltrative growth pattern, high nuclear grade, high cellularity, necrosis, mitotic activity > 1/50 HFP and vascular invasion. Thereby, PEComa with two or more worrisome histologic features should be considered as malignant. Tumors with nuclear neoplasm only or size more than 5 cm only were considered as a neoplasm of uncertain malignant potential. Only one case of malignant PEComa was reported in the liver [9], in which the diagnosis was established based on the presence of metastasis. The microscopic findings, in this case, were consistent with a benign status except for size more than 5 cm. Seven were thought to have malignant potential according to Folpe’s criteria, but did not exhibit recurrence or metastasis. This might be attributable to the fact that the follow-up durations in these cases were relatively short (3 to 24 month). In our case the patient exhibited two features that favored malignancy, tumor size and infiltrative growth pattern, requiring therefore, a close and long-term follow-up.

There is no specific treatment protocol for hepatic PEComa; the most of neoplasm were surgically treated without any adjuvant therapy.

## Conclusion

Primary PEComas of the liver are still a curiosity and therefore the diagnostic approach, treatment modalities and the follow-up are challenging. Their prognosis remains unpredictable; it should be regarded as tumors with uncertain biological potential that require strict and long-term follow-up.

## Consent from the patient

Written informed consent was obtained from the patient for publication of this case report and any accompanying images.

## Abbreviations

PEComas: Perivascular epithelioid cell neoplasms; PECs: Perivascular epithelial cells; NOS: Not otherwise specified; AFP: Alpha-foetoprotein; CEA: Carcinoembryonic antigen; CT: Computed tomography; MRI: Magnetic resonance imaging; Fig: Figure; HMB45: Human melanoma black 45; SMA: Smooth muscle actine; AML: Angiomyolipoma; TSC: Tuberous sclerosis complex.; GIST: Gastrointestinal stromal tumor.

## Competing interests

The authors declare that they have no competing interests.

## Authors’ contributions

All authors read and approved the final manuscript.

## Supplementary Material

Additional file 1: Table S1 Clinical-characteristics, radiographic and pathologic features, and the follow up of previously reported cases of hepatic PEComas-NOS. Click here for file

## References

[B1] Fletcher CD Unni KK Mertens F World Health Organization Classification of Tumors of Pathology and genetics of soft tissue and bone 2002 Lyon, France: IARC Press 221 222

[B2] Zamboni G Pea M Martignoni G Zancanaro C Faccioli G Gilioli E Pederzoli P Bonetti F Clear cell “sugar” tumor of the pancreas: a novel member of the family of lesions characterized by the presence of perivascular epithelioid cells Am J Surg Pathol 1996 20 722 730 8651352 10.1097/00000478-199606000-00010

[B3] PubMedhttp://www.ncbi.nlm.nih.gov/pubmed/

[B4] Bonetti F Pea M Martignoni G Zamboni G PEC and sugar Am J Surg Pathol 1992 16 307 308 1599021 10.1097/00000478-199203000-00013

[B5] Folpe AL Kwiatkowski DJ Perivascular epithelioid cell neoplasms: pathology and pathogenesis Hum Pathol 2010 41 1 15 19604538 10.1016/j.humpath.2009.05.011

[B6] YU D Tang S Hepatic perivascular epithelioid cell tumor: a case report and review of the literature Inter Med 2013 52 1333 1336 10.2169/internalmedicine.52.0144 23774542

[B7] Yamasaki S Tanaka S Fujii H Matsumoto T Okuda C Watanabe G Suda K Monotypic epithelioid angiomyolipoma of the liver Histopathology 2000 36 451 456 10792487 10.1046/j.1365-2559.2000.00848.x

[B8] Tryggvason G Blondal S Coldin RD Albrechtsen J Bjornsson J Jonasson JG Epithelioid angiomyolipoma of the liver: case report and review of the literature APMIS 2004 112 612 616 15601311 10.1111/j.1600-0463.2004.apm1120909.x

[B9] Parfitt JR Bella AJ Izawa JI Wehrli BM FRCPC Malignant Neoplasm of Perivascular Epithelioid Cells of the Liver: late widespread metastasis with long-term follow-up Arch Pathol Lab-Med 2006 130 1219 1222 16879028 10.5858/2006-130-1219-MNOPEC

[B10] Fang SH Zhou LN Jin M Hu JB Perivascular epithelioid cell tumor of the liver: a report of two cases and review of the literature World J Gastroenterol 2007 13 5537 5539 17907305 10.3748/wjg.v13.i41.5537 PMC4171296

[B11] Larbcharoensub N Karnsombut P Jatchaval J Wasutit Y Nitiyanant P Primary hepatic clear cell myomelanocytic tumor: case report and review of the literature APMIS 2007 115 1454 1459 18184420 10.1111/j.1600-0463.2007.00733.x

[B12] Svajdler M Bohus P Goe V Tkacova V Perivascular Epithelioid Cell Tumor (PEComa) Cesko slovenska pathologie 2007 43 18 22 17370472

[B13] Zimmermann A Von der Brelle C Berger B Kappelr A Candinas D Primary perivascular epithelioid cell tumor of the liver not related to hepatic ligaments: hepatic PEComa as an emerging entity Histopathology 2008 23 1185 1193 10.14670/HH-23.1185 18712670

[B14] Paiva CE Moraes Neto FA Agaimy A Domingues AC Rogatto SR Perivascular epithelioid cell tumor of the liver coexisting with a gastrointestinal stromal tumor World J Gastroenterol 2008 14 800 822 18205275 10.3748/wjg.14.800 PMC2684012

[B15] Strzelczyk JM Durczynski A Szymanski D Jablkowski M Dworniak D Sporny3 Sriola. Perivascular epithelioid cell tumors (PEComa) of the liver: report of case Surg Today 2009 39 916 921 19784736 10.1007/s00595-009-3945-5

[B16] Priola SM Cataldi A Marci V Fava S Acute abdomen as an unusual presentation of hepatic PEComa: A case report Tumori 2009 95 123 128 19366072 10.1177/030089160909500124

[B17] Perez SB Tumor de cellulas epithelioides perivasculares (PEComa) hepatico CIR ESP 2009 85 179 189 19264299 10.1016/j.ciresp.2008.09.010

[B18] Akitake R Kimura H Sekoguchi S Nakamura H Seno H Chiba T Fujimoto S Perivascular epithelioid cell tumors (PEComa) of the liver diagnosed by contrast-enhanced ultrasonography Inter Med 2009 48 2083 2086 10.2169/internalmedicine.48.2133 20009396

[B19] Wen MC Jan YJ Li MC Wang J Lin A Monotypic epithelioid angiomyolipoma of the liver with TFE3 expression Pathology 2010 42 300 302 20350230 10.3109/00313021003631254

[B20] Ahn JH Bang H Primary perivascular epithelioid cell tumor (PEComa) of the liver: A case report and review of the literature Korean J Pathol 2011 45 93 97 10.4132/KoreanJPathol.2012.46.5.499 PMC3490117 23136579

[B21] Jafari A Fischer HP Websky MV Hong GS Kalff JC Manekeller SZ Primary perivascular epitheloid cell tumour (PEComa) of the liver: case report and review of the literature Gastroenterol 2013 51 1096 1100 10.1055/s-0033-1350123 24022205

[B22] Cheung TT Smith NT Poon RTP Primary perivascular epithelioid cell tumor (PEComa) of the liver BMJ Case Rep 2013 doi:10.1136/bcr-2013-008706 10.1136/bcr-2013-008706 PMC3736252 23845671

[B23] Zhao LJ Yang YJ WU H Huang SM UU K Perivascular epithelioid cell tumor of the liver: a case report and literature review Eur Rev Med Pharmacol Sci 2013 17 1665 1668 23832736

[B24] Patra S Vij M Kota V Kancherla R Rela M Pigmented perivascular epithelioid cell tumor of the liver: Report of a rare case with brief review of literature J Can Res Ther 2013 9 305 307 10.4103/0973-1482.113401 23771383

[B25] Sheng HQ Chen DF Sun XH Li X Xu J Hu XB Li MQ Zhang RY Li KZ MRI diagnostic of perivascular epithelioid cell tumor (PEComa) of the liver Rom J Morphol Embryol 2013 54 643 647 24068418

[B26] Folpe AL Mentzel T Lehr HA Fisher C Balzer BL Weiss SW Perivascular epithelioid cell neoplasm of soft tissue and gynecologic origin: a clinicopathologic study of 26 cases and review of the lliterature Am J Surg Pathol 2005 29 1558 1575 16327428 10.1097/01.pas.0000173232.22117.37

