# Supplementary material for: Primary perivascular epithelioid cell tumor of the liver: new case report and literature review
Source: Diagn Pathol. 2014 Jul 17;9:149. doi: 10.1186/1746-1596-9-149 (PMC4223599; doi:10.1186/1746-1596-9-149)
Supplement: Additional file 1: Table S1 — Clinical-characteristics, radiographic and pathologic features, and the follow up of previously reported cases of hepatic PEComas-NOS. [file 1746-1596-9-149-S1.docx]

**Table S1: Clinical-characteristics, radiographic and pathologic features, and the follow up of previously reported cases of hepatic PEComas-NOS**

| **Authors & years** | **Age  y/ Sex** | **Medical history** | **Site** | **Size**  **(cm)** | **Imaging appearance** | **Pathological features** | **Immunohistochemical staining** | **Treatment** | **Follow up** | **Event** |
| --- | --- | --- | --- | --- | --- | --- | --- | --- | --- | --- |
| **Yamasaki S and *al* 2000 ^6^** | 30/F |  | Right lobe | 3 | -CT: Hypodense mass | -Polygonal epithelioid cells with a rich network of sinusoidal blood vessels.  -Focal pleomorphism with frequent multinucleate cells  -Mitoses absente.  -Focal Haemorrhagic necrosis and Venous invasion.  -Focal cystic degeneration | -Positivity for HMB45& S100 protein  -Focal positivity for vimentin, desmin and SMA.  -CK, AFP, chromogranin A, synaptophysin, Factor  VIII and EMA were negative. | Partial hepatectomy | 12 months | NO |
| **Trygvason G and *al*  2004  ^7^** | 42/F |  | Left lobe | 7 | CT: well demarcated masse with significant and heterogeneous enhancement on arterial phase.  The mass turn isotense to the liver in portal phase. | -Sheets of larges epithelioid cells, with clear abundant cytoplasm and proeminent perinuclear condensations.  -Mild nuclear pleomorphism.  -Mitoses absent. | -Strong and diffuse positivity for HMB45&Melan A.  -EMA, CEA, CD117, desmin, SMA, chomogranin and S100 protein were negative | Resection of segment II&III. | 23 months | NO |
| **Parfitt JR and *al* 2006 ^8^** | 60/F |  | Right lobe | 14 | CT: Tumoral mass in the right liver | -Sheets and nests of epithelioid and spindled cells, with abundant eosinophilic to clear cytoplasm.  -Round nuclei with little pleomorphism and occasional nuclear inclusions.  -Mitoses rare  -Necrosis: discrete. | -Strong and diffuse positivity for HMB45, Melan A and SMA.  -CD117, desmin: weak and focal.  - S100 protein, CK, CK8/18, CEA, CD31, CD34, CD45, factor VIII, vimentine, chomogranin, synapatophysin and myoglobin were negative | Right hepatic lobectomy | 10 years | Hepatic recurrence, and pulmonary, pancreatic and muscular metastases (neck) at 9 years.  - Bladder metastases at 10 years.  -Alive with disease at 10 years. |
| **Fang and *al* 2007  ^9^** | 56/F |  | Left lobe | 5,1 | Contrast-enhanced CT: significant enhancement more striking on portal venous phase than arterial phase | -Polygonal or short spindle cells with clear abundant cytoplasm | Positivity for HMB45, Melan A |  | 24 months | NO |
| **Larbcharoensub N and *al*  2007 ^10^** | 31/F |  | Segment VIII | 1,8 | -MRI: Well circumscribed hyposignal T1W/ iso to slightly hypersignal T2W.  -After gadolinium: homogenous early arterial phase with rapid washout in the portal phase. | -Round to polygonal cells with abundant clear cytoplasm and distinct cell borders.  -Mitoses, necrosis and angiolymphatic invasion were absent | - Positivity for HMB45, Melan A, HHF35 and vimentine.  - SMA, S100 protein, CK, Desmin, H-caldesmon, CEA, CD10, CD31, CD34, CD117, factor VIII, chomogranin, synapatophysin and hormonal receptors were negative  -Ki67<1%. | Partial hepatectomy | 6 months. | NO |
| **Svajdler M and *al* 2007 ^11^** | 55/F | Glioblastoma treated with chemoradiotherapy | Left lobe | 3,5 |  | -Sheets of clear and eosinophilic epithelioid cells.  -Mild nuclear pleomorphism.  -Mitoses: 5/50 HPF.  -Vascular invasion & Necrosis : absent | - Positivity for HMB45, Melan A, and SMA  -Sporadic nuclear positivity for P53 (1%)  -S100 protein, HHF35, CK, EMA, vimentine, desmin, CD10, TTF1, cyclinD1 were negative  -Ki67: 3-5 % | Complete resection of tumor |  |  |
| **Zimmermann A and *al* 2008 ^12^** | 53/M |  | Segments V&VIII | 8 | MRI:  - T2 emphasis: The central region of tumor is hyperintense with small areas of liquid collection, whereas the periphery appears hypointense.  -Contrast enhanced T1: The lesion displays a heterogeneous intensity with a strong enhancement in the central part of the tumor. | -Nests, bundles and tiny nodules of epithelioid and spindle cells, forming, at some places, palisade- like structures.  -Some cells had a finely granular dark brown and iron-negative pigment.  -Mild nuclear pleomorphism with central necrosis.  -Focal lymphocytic & plasmocytic infiltrating. | -Markedly reactivity for HMB45, Melan A, PLN2 and Béta cathenine.  -SMA reactivity was chiefly involved the slender, myeloid looking spindle cells arranged in bundles.  - S100 protein, thyrosinase, CK, vimentine, desmin, myogenin, CD34 CD 117 were negative  -Ki67<1%. | Right hemihepatectomy | 17 months. | NO |
| **Paiva CE and *al***  **2008 ^13^** | 51/F | -Uterine leiomyomatosis (2006).  -Synchronous gastrique GIST | Left lobe | 0,8 |  | -Sheets of epithelioid cells with abundant eosinophilic or clear cytoplasm  -Numerous dilated vascular spaces with hemangiopericytic pattern  -Mild nuclear pleomorphism without mitosis or necrosis. | -Positivity for HMB45, HHF35, and vimentine  -S100 protein, CK, CD34 and CD117 were negative. | Complete resection of tumor | 25 months. | NO |
| **Strzelczyk JM and *al* 2009 ^14^** | 57/F |  | Right lobe | 17 | CT: Cystic mass in the right lobe | -Oval and polygonal  clear or slightly eosinophilic cytoplasm  -Focal nucleus polymorphism  -Mitoses : 1-2/20 HPF. | -Strong positivity for HMB45  -Moderate positivity S100 protein and SMA.  -CK, CK7, CK9, CK20, EMA, CD31, CD34, desmin, chromogranin A, synaptophysin and factor VIII were negative. | Right hemihepatectomy | 53 months. | NO |
| **Priola AM and *al* 2009 15** | 36/F |  | Left lobe | 11 | -Precontrast CT image: inhomogeneous and hypoattenuating lesion.  -Contrast enhanced CT: intense and homogenous enhancement of the lesion on arterial phase. Ring enhancement persists in the last phase. | -Monotonous growth in trabecular pattern of clear spindle cells with finely granular cytoplasm.  -Mitoses<3/10 HPF.  -Diffuse areas of hemorrhage, cystic changes and abundant central necrosis | -Strong positivity for HMB45, Melan A and SMA.  -Weak positivity for vimentine  -S100 protein, CD34, AFP, CK and hepatocytic-specific antigen were negative | Left hepatic lobectomy | 34 months. | NO |
| **Perez SB 2009 ^16^** | 32/F |  | Segment VII | 5,5 | -CT contrast: heterogeneous enhancement with central hypodensity.  -MRI: Well circumscribed hypersignal T2W. | Trabecular pattern of epithelioid perivascular cells. | -HMB45 and SMA were positive.  -S100 protein and Desmin were negative | Median Segmentectomy (VII) |  |  |
| **Akitake R and *al* 2009 ^17^** | 36/F | Bronchial asthma | Segment II | 3,5 | -Contrast enhanced ultrasonography: tumor was enhanced in early arterial phase, and the reagent rapidly flowed into drainage veins.  - Non-enhanced CT: Low density tumor.  -Non-enhanced MRI: Low intensity on T1-weighted image, and high intensity on T2- weighted image.  -Fat suppressive MRI did not show suppression of hyperintensity of the tumor. | -Highly cellular tumor consisting of fascicules of larges polygonal cells with abundant cytoplasm.  -Rare mitosis. | -Strong and diffuse expression of HMB45.  -Partial expression of SMA.  -CK, CD34 and S100 protein were negative. | Lateral segmentectomy | 18 months. | NO |
| **Wen MC and *al***  **2010 ^18^** | 25/M | Mediastinal large B cell lymphoma treated with chemotherapy 2years ago | Segment IV | 5 | -CT scan: hyperdense lesion.  -Enhanced-MRI: hypervascular mass with early enhancement and rapid washout in the dynamic MRI. | -Il-defined proliferation of larges and polygonal cells with distinct borders and Eosinophilic cytoplasm.  -Oval nuclei with prominent nucleoli.  -Dark brown melanin pigments were focally noted.  -Mitotic figures were scanty.  -Small foci of coagulative necrosis.  -Vascular and lymphatic invasion | -Diffuse expression of HMB45.  -Strong nuclear expression for TFE3.  -Focal expression of vimentin and S100 protein.  -Melan A, CK, EMA, hepatocyt antigen, synaptophysin, chromogranin A, CD10, actin and desmin were negative.  -Ki67: 10%. | Hepatic segmentecomy |  |  |
| **Ahn JH and *al***  **2011 ^19^** | 36/F |  | Left lateral segment | 7 | CT scan: Well-defined mass with hetergeneous signal intensity.  Multiple tiny cysts in both lobes. | -Unencapsulted tumor that infiltrates adjacent parenchymal liver tissue.  -Polygonal epithelioid and spindle cells with sheets and vaque trabecular pattern.  -Rich sinusoidal vascular network.  -Presence of dark or light brown pigment.  -Absence of cellular pleomorphism,  -Absence of necrosis and vascular invasion | -Strong expression of HMB45 and SMA  -Weakly reactivity for S100 protein.  -Melan A positive in epithelioid cells.  -CK, CD34, and CD117 were negative  -Ki67: 1%. | Lateral segmentectomy | 3 months. | NO |
| **Jafari A and al**  **2013 ^20^** | 53/F |  | Segment II&III | 7,5 | -Enhancement CT: hyper vascularized tumor.  -MRI: hyposignal T1W/ inhomogeneous hypersignal T2W.  -After gadolinium: intensive early arterial contrast enhancement with a rmoderate washout phenomenon in the later following sequences. | -Epithelioid cells with amphiphilic to eosinophilic cytoplasm.  -Areas of alveolar to adenoid formation. | -Diffuse expression of Melan A.  -Focal expression of HMB45 and S100 protein.  -HEPAR1 was negative.  -Ki67: 2%. | Left lateral bisegmentecomy | 14 months. | NO |
| **Cheung TT and *al* 2013 ^21^** | 53/F |  | Right lobe | 10 | -CT: hypervascular tumor with arterial enhancement at postcontrast image and “washout” pattern in the delayed portal venous phase. | -Sheets of epithelioid and spindle cells of moderate cellularity.  -Perivascular arrangement of spindle cells. | -Positivity of HMB45 and Melan A  -Hepatocytic markers, CD31, CD21, and desmin were negative | Right hepatectomy |  |  |
| **ZHAO LJ and *al* 2013 ^22^** | 58/M |  | segment IV | 7,6 | -CT: heterogeneous and lower density lesion with obvious enhancement on arterial phase and slight hypodensity on portal phase. | -Epithelioid and spindle cells with eosinophilic cytoplasm arranged disorganized.  -Many lymphocytes and fibroblasts/myofibroblasts were invaded in the neoplasm. | -Positivity of HMB45 and MART1  -Focal positivity for SMA and ALK1.  -CK, S100 protein, EMA, Heppar-1 were negative | Right IV segmentecomy |  |  |
| YU D **and *al* 2013 ^23^** | 41/F |  | Segment VI | 1,5 | -CT: hypointense nodular lesion with peripherally enhancement during the arterial phase. The degree of enhancement then decreased during the portal phase. | -Pleomorphic round to polygonal large cells with abundant eosinophilic and granular cytoplasm.  -Bundles of smooth muscle.  -Rare Mitotic figures. | Positivity of HMB45 and Melan A | Right hepatic lobectomy | 9 months. | NO |
| **Patra S and *al***  **2013 ^24^** | 50/F |  | Segment VIII | 10 | CT: large tumor in segment VIII with multiples hemangiomas in both the lobe of the liver. | -Sheets and nests of spindle and epithelioid cells with large deposits of brown pigments (melanin)  -Focal nuclear pleomorphism.  -Presence of cystic areas. | -Strong and diffuse positivity for HMB45.  - Focal positivity for SMA.  -Heppar-1, Glypican 3, CK, desmin, MUM-1 and S100 protein were negative | Complete resection of tumor. | 24 months. | NO |
| **Sheng HQ and al 2013 ^25^** | 55/M |  | Segment VI | 1,6 | -CT: homogeneous hypoattenuating lesion.  -MRI: Hypointense on T1-weighted images. Hypointense and hyperintense areas on T2-WI, and hyperintense on diffusion-weigthed images, without fat in the fat tissue suppression sequence.  -Dynamic MRI after injection of gadolinium: homogeneous enhancement during the arterial phase, rapidly attenuated during de portal phase, with hypoenhancement in the late parenchymal phase | -Sheets and nests of epithelioid and some spindled cells with clear to eosinophilic cytoplasm. | - Positivity for HMB45, Mela A and SMA. | Complete resection of tumor. | 12 months. | NO |
| **Present case** | 63/F |  | Segment IV | 8 | -CT: large and hypointense tumor in segment IV. After injection of contrast product, the lesion showed heterogeneous enhancement that persists in portal phase.  -MRI: low signal on T1-Weighted images, it became hyperintense on T2-Weighted images, and presented a strong and heterogeneous enhancement after injection of gadolinium. | -Il-defined proliferation of larges and polygonal cells with distinct borders and Eosinophilic to clear cytoplasm.  -Oval nuclei with prominent nucleoli.  -Mitotic figures: absente | -Strong and diffuse positivity for HMB45 and SMA  - Focal positivity for Melan A.  - CK and S100 protein were negative | Hepatic segmentectmy | 9 months | NO |

CT : Computerized tomography, MRI : Magnetic resonnance imaging, WI: Weighted images, EMA : epithelial membran antigen, CEA : carcinoembryonic antigen, SMA : smooth muscle actin, HHF35: muscle specific actin, AFP: alpha fetoprotein, HPF: high power fields.
